# Early Feeding Strategy Mitigates Major Physiological Dynamics Altered by Heat Stress in Broilers

**DOI:** 10.3390/ani14101485

**Published:** 2024-05-16

**Authors:** Ahmed Gouda, Hanan Al-Khalaifah, Afaf Al-Nasser, Nancy N. Kamel, Sherin Gabr, Kamal M. A. Eid

**Affiliations:** 1 Department of Animal Production, National Research Center, El Buhouth St., Dokki, Giza P.O. Box 12622, Egypt; 2 Environment and Life Sciences Research Center, Kuwait Institute for Scientific Research (KISR), P.O. Box 24885, Safat, Kuwait City 13109, Kuwait; hkhalifa@kisr.edu.kw (H.A.-K.); anasser@kisr.edu.kw (A.A.-N.); 3 Department of Poultry Breeding Research, Animal Production Research Institute, Ministry of Agriculture, Dokki, Giza P.O. Box 12611, Egypt; sherygabr7@gmail.com (S.G.); kamaleid2003@yahoo.com (K.M.A.E.)

**Keywords:** heat stress, feed withdrawal, broiler, performance, metabolic hormones, immune responses, redox status, HSP70

## Abstract

**Simple Summary:**

In order to ensure the profitability of the broiler industry, it is essential to maintain optimal performance, especially under undesirable environmental conditions. Elevation in environmental temperatures and its subsequent negative impacts on broilers’ physiological and metabolic homeostasis deleteriously affect production performance. Early adaptation is an effective strategy for ensuring sustainable broiler production. We investigated three early feed withdrawal (FWD) regimes for 24 h, either continuous or equally distributed over two or three days, as potential thermal stress-mitigating strategies. The results demonstrated a positive adjustment in metabolic hormones and biochemical metabolite markers in addition to an elevation in antioxidant enzyme activity and immune response in the FWD groups. Finally, we established that the investigated FWD strategies can promote broiler thermotolerance adaptation, reflected in the significant enhancement in broiler production performance, immunomodulation response, and recovery of the antioxidant balance.

**Abstract:**

Heat stress is one of the stressors that negatively affect broiler chickens, leading to a reduction in production efficiency and profitability. This reduction affects the economy in general, especially in hot and semi-hot countries. Therefore, improving heat tolerance of broiler chicks is a key to sustained peak performance, especially under adverse environmental heat stress conditions. The present study investigated three early feed withdrawal regimes (FWD) as a potential mitigation for thermal stress exposure. A total of 240 unsexed one-day-old Cobb-500 chicks were randomly recruited to one of four experimental groups using a completely randomized design (10 birds × 6 replicates). The experimental groups included the control group with no feed withdrawal (control), while the other three groups were subjected to early feed withdrawal for either 24 h on the 5th day of age (FWD-24), 12 h on the 3rd and 5th day of age (FWD-12), or 8 h on the 3rd, 4th, and 5th day of age (FWD-8), respectively. Production performance was monitored throughout the experiment. Meanwhile, blood and liver samples were taken at the end of the experimental period to evaluate major physiological dynamic changes. Our findings demonstrated that under chronic heat stress conditions, FWD treatments significantly improved broilers’ production performance and enhanced several physiological parameters compared with the control. Serum levels of thyroid hormones were elevated, whereas leptin hormone was decreased in FWD groups compared with the control. Moreover, serum total protein, globulin, and hemoglobin levels were higher, while total cholesterol and uric acid were lower in the FWD groups. Furthermore, FWD groups showed significantly higher antioxidant marker activity with a significantly lower lipid peroxidation level. Immunoglobulin levels, lysozyme, complement factor C3, and liver heat shock protein 70 (HSP70) concentration were also elevated in FWD compared with the control. Also, serum interleukin-1β (IL-1β) and interferon-gamma (IFN-γ) significantly increased with FWD. Based on our findings, early feed withdrawal can be applied as a promising non-invasive nutritional strategy for broilers reared under chronic heat stress conditions. Such a strategy promotes the alleviation of the deleterious effects of heat stress on broiler performance, immunity, and redox status, owing to the onset of physiological adaptation and the development of thermotolerance ability.

## 1. Introduction

Broiler chicken production is a vital sector of the global poultry industry, providing a major protein source for human nutrition [1]. However, the optimum productivity of broilers is often hindered by various environmental stressors. In tropical and subtropical regions, heat stress is considered a major challenge confronting the modern poultry industry as it can compromise broilers’ growth rate, immune response, redox status, and the overall welfare of the birds [2,3,4,5]. High temperatures, coupled with humidity, can result in heat stress, which disrupts normal physiological processes and impairs the performance of broiler chickens [6]. Awad et al. [7] stated that the deleterious effects of heat stress on broilers’ performance and immune responses were consistent across different commercial strains. Thus, overcoming such a challenge is essential for obtaining sustainable poultry meat production that covers the growing global demand. Early-life adaptation was imposed as a potentially potent strategy to mitigate the negative consequences of stress exposure during the birds’ lifespan [8,9,10].

In recent years, researchers have explored innovative strategies to mitigate the adverse effects of heat stress and recover broiler performance, immunity, and redox balance. Strategies to mitigate the negative impacts of heat stress are of paramount importance for the sustainable and efficient production of broilers [11]. Feeding strategies targeting increased dietary intake, reduced heat production, and improved general health are highly necessary for the nutritional management of chickens under heat stress [12]. Wet feeding, feeding form, feeding structure, feed additives, dual feeding, and feed restriction were some of the promising strategies proposed to reduce the deleterious effects of heat stress on poultry production [13,14,15,16,17,18,19,20]. One such promising strategy that has gained attention recently is feed withdrawal at an early age. Previous research has explored the theory of applying mild stress at early ages to potentially mitigate the negative effects of stress exposure later in life. These programs include thermal conditioning and feed manipulation techniques, like withdrawal or restriction. El-Moniary et al. [21] suggested that feed withdrawing for 24 h on the fifth day of age could improve the productivity of broiler chicks under summer stress conditions. Early feed withdrawal has emerged as a potential solution to combat heat stress in poultry [22]. This practice involves temporarily restricting the access to feed during the critical early stages of broiler development, with the goal of enhancing their ability to cope with heat stress exposure later in life [23]. This approach is rooted in the idea that early nutritional management can have lasting effects on the birds’ thermotolerance and overall performance under challenging environmental conditions [12,24]. Zhan et al. [25] suggested a prolonged metabolic programming induction in broiler chicks exposed to early feed restriction for four hours a day during the first 21 days of age. Accordingly, early feed withdrawal can be used to induce broilers’ heat tolerance ability through an early metabolic adaptation, which consequently optimizes nutrient utilization and improves performance during heat stress exposure. The optimal and effective feed withdrawal regimes under thermal stress and their potential for physiological adaptation are not yet fully defined.

The present research was designed to clear out and provide an in-depth exploration of feed withdrawal at an early age as a potential nutritional intervention to reduce the deleterious influences of heat stress on broilers’ production, immunological parameters, and redox status. We aimed to contribute valuable insights to the ongoing efforts to enhance the resilience and productivity of broiler chickens facing the challenges of heat stress through investigating the effectiveness and the mechanisms underlying early feed withdrawal at different intensities to enhance broiler resilience and induce thermotolerance.

## 2. Materials and Methods

### 2.1. Bird Management and Experimental Design

Two hundred and forty unsexed one-day-old Cobb-500 chicks were randomly divided into four experimental groups using complete randomized design (10 birds × 6 replicates). After distributing the birds, the sex ratio in each group replicate was found to be 1 male to 9 females. Chicks were reared in a hierarchically designed cage system divided by wire mesh barriers with dimensions of 1.0 m × 0.50 m × 0.40 m. Each compartment housed ten birds and was identical to the others. The experimental groups were the control group with no feed withdrawal (control), while the other three groups were subjected to early feed withdrawal for either 24 h on the 5th day of age from 7:00 a.m. to 7:00 a.m. next day (FWD-24), 12 h on the 3rd and the 5th day of age from 7:00 a.m. to 7:00 p.m. (FWD-12), or 8 h on the 3rd, 4th, and 5th day of age from 7:00 a.m. to 3:00 p.m. (FWD-8), respectively. Afterward, all experimental birds were fed ad libitum with free access to water until the end of the experiment. The experimental diets were formulated to cover the nutrition requirements of broilers, following the recommendation of NRC [26] and the Cobb-500 broiler management guide with two formulas: for starter (from 1–21 days of age) and grower–finisher (from 22–35 days of age) (Table 1). All birds were housed under the same management conditions. The lighting regime was set at 23 h light and 1 hr dark for the first three days of the experiment; afterward, it was adjusted to 16 h light and 8 h dark for the rest of the experiment period.

### 2.2. The Experimental Environment Conditions

The present study was carried out during the summer season. Throughout the experimental period, the minimum and maximum ambient temperatures as well as humidity percentage were monitored every day, and the average weekly readings were then calculated. Accordingly, the corresponding temperature–humidity index (THI) was calculated [27]. The average weekly changes in ambient temperatures, relative humidity percentage, and the calculated THI are presented in Table 2. The THI value ranged from 28.3 to 33.8, indicating that birds were subjected to chronic heat stress conditions.

### 2.3. Production Performance

Chicks were individually weighed on the 1st, 21st, and 35th day of age and weight gain (BWG) was then calculated. Meanwhile, feed intake (FI) was recorded on a replicate basis at the end of the starter period and the finisher period. Accordingly, feed conversion ratio (FCR) was measured as g feed intake per g weight gain.

### 2.4. Blood Samples Collection 

At end of the experimental period, blood samples were collected from each experimental group as one sample per each group replicate (n = 6, all from females). For serum collection, blood samples were taken in clean and dry vials and centrifuged at 4000 rpm for 15 min at room temperature. The collected serum was stored at −20 °C until further analysis. Another fresh blood sample (n = 6, all from females) was used to estimate blood hemoglobin (Hb) according to the method described by Jain [29].

### 2.5. Serum Metabolic Hormones and Biochemical Analysis

Serum thyroid hormone concentrations, triiodothyronine (T_3_), and thyroxin (T_4_), were assessed using commercial Radio immunoassay (RIA) kits (Byk-Sangtec Diagnostica, Dietzenbach, Germany, Immulite 2000, DPC, Los Angeles, CA, USA), following the method of Sánchez-Carbayo et al. [30]. Serum levels of leptin were measured using chicken specific ELISA kit (MBS025331; MyBiosource, San Diego, CA, USA). Serum total protein (TP) and albumin concentrations were measured according to the methods of Weichselbaum [31] and Doumas et al. [32], respectively. Meanwhile, globulin was mathematically calculated by subtracting serum albumin from serum TP values. Serum total cholesterol was measured according to Allain et al. [33]; uric acid was measured according to Sanders et al. [34], using an automatic biochemical analyzer (Robonik Prietest ECO, Ambernath (West), Thane, India).

### 2.6. Serum Antioxidant Markers

Serum total antioxidant capacity (TAC) was determined according to the method of Janaszewska and Bartosz [35]. Catalase (CAT) and superoxide dismutase (SOD) enzyme activities were assessed according to the methods of Aebi [36] and Sun et al. [37], respectively. Malondialdehyde (MDA) concentration was determined according to the method described by Placer et al. [38].

### 2.7. Immunological Parameteres 

Blood serum immunoglobulin (Ig), IgA, IgG, and IgM, levels were determined using indirect ELISA kits (MyBio-Source, Inc., San Diego, CA, USA) according to Tiemann et al. [39]. The serum lysozyme activity was determined according to Lie et al. [40]. The serum level of complement 3 protein (C3) was determined using a sandwich enzyme-linked immunosorbent assay (ELISA) kit (LS-F9287; LifeSpan Biosciences, Inc., Seattle, WA, USA). To quantify interleukin 1β (IL-1β) and interferon gamma (INF-γ), chicken-specific ELISA assay kits were used (MBS2024496 and MBS2024496, respectively; MyBioSource, San Diego, CA, USA).

### 2.8. Liver Heat Shock Protein 70 

To induce the production of heat shock protein 70 (HSP70), birds were subjected to 42 °C for one hour before slaughter [41]. Afterwards, the liver was instantly dissected out (n = 6, all from females) and then vacuum packed and kept at −20 °C. The HSP70 was measured using ELISA following the method of Anderson et al. [42].

### 2.9. Statistical Analysis 

The data underwent one-way analysis of variance (ANOVA) utilizing IBM SPSS Statistics 20 (IBM Corp., Chicago, IL, USA). Replicates (n = 6) served as the experimental unit for production performance parameters, while the individual bird was the experimental unit for the physiological parameters. Group means were assessed for significant differences using Duncan’s multiple range test at a confidence level of 95% (*p* ≤ 0.05). The findings were reported as mean ± standard error of the mean (SEM).

## 3. Results

### 3.1. Production Performance

Table 3 presents the growth performance of broiler chicks under various early feed withdrawal (FWD) treatments. The results highlight the impact of different FWD strategies on key growth parameters. The initial body weights (BW) of the chicks across all treatments were similar, with no significant differences observed. Meanwhile, BW at 21 days varied significantly among treatments. Treatment FWD-24 had the highest BW and was significantly different from other experimental groups. Furthermore, treatment FWD-24 resulted in the highest final BW, followed by FWD-8 and FWD-12, while the control had the lowest weight. The increased level of final BW compared with the control group was 20, 9, and 13% for FWD-24, FWD-12, and FWD-8, respectively. Consistently during the period from 1 to 21 days of age, FWD-24 led to the highest body weight gain (BWG), followed by FWD-8 and FWD-12. The same significantly higher BWG persisted for the overall experimental period 1–35 days of age, with FWD-24 showing the highest BWG, followed by FWD-8 and FWD-12, respectively. Meanwhile, the results of feed intake (FI) showed no significant differences during the starter period from 1 to 21 days of age across treatments. However, during the period from 22 to 35 days of age and for the overall experimental period, FWD groups had a significantly higher FI than that of the control group. Accordingly, the FWD-24 group had the best FCR through the experimental period, followed by FWD-8 and FWD-12 groups, respectively, compared with the control group. The current results indicate that early feed withdrawal strategies significantly influenced the growth performance of broiler chicks. Treatment FWD-24, with feed withdrawal for 24 h on the fifth day of age, resulted in the highest body weights and most favorable FCR in various growth periods.

### 3.2. Blood Metabolic Hormones and Biochemical Markers

The metabolic-related hormones concentration of broilers subjected to early feed withdrawal (FWD) strategies are presented in Table 4. Triiodothyronine (T_3_) and thyroxin (T_4_) levels showed a higher concentration for the FWD groups compared with the control group. The FWD-24 group had the highest T_3_ and T_4_ concentration by 12 and 13%, followed by FWD-8 by 9 and 9%, and finally FWD-12 by 5 and 8%, respectively. Meanwhile, the FWD groups showed a significant reduction (24–32%) in leptin concentration, an appetite regulation hormone, compared to the control group.

The impact of various early feed withdrawal (FWD) strategies on blood serum metabolites concentration is presented in Table 4. The results demonstrate significant differences among treatments, indicating the influence of FWD on metabolic parameters. A significantly higher serum total proteins (18–24%), globulin (27–31%), and Hb (10–12%) levels were observed for the FWD treatment groups compared with the control group. Contrarily, serum total cholesterol and uric acid showed significantly lower levels for the FWD treatment groups, ranging from 7 to 10% and 10 to 11%, respectively. Thus, it seems that early feed withdrawal strategies significantly enhanced thyroid hormone levels and ameliorated blood serum metabolite concentrations while decreasing the level of leptin hormone concentration in heat-stressed broiler chickens. These findings offer insights into the metabolic adaptation responses of broilers to different early feed withdrawal practices.

### 3.3. HSP70 and Oxidation Markers

The heat shock protein 70 (HSP70) and redox status markers of different early feed withdrawal (FWD) strategies are presented in Table 5. The results reveal significant differences in the investigated antioxidant markers and HSP70 level among the different experimental groups. Treatment FWD-24 showed the highest HSP70 levels, followed by FWD-12 and FWD-8, while the control had the lowest level. The fold increase in HSP70 levels for the FWD groups was approximately 1.8-fold higher than that observed in the control group. Meanwhile, the redox status improved significantly in response to various FWD treatments. The total antioxidant capacity (TAC), catalase, and superoxide dismutase (SOD) activities were significantly higher for the FWD groups by approximately 1.3, 1.9, and 1.1-fold, respectively, compared with the control group. Meanwhile, the MDA level, an oxidative stress indicator marker, was significantly lower by 45 to 50% in the FWD groups compared with the control group. Eventually, the antioxidant status of broiler chickens was significantly improved by different early feed withdrawal strategies. These findings have implications for broiler general health and can guide optimal feeding practices under heat stress challenges.

### 3.4. Innate and Humoral Immuneity Marker Levels

Table 6 provides results related to the effect of various early feed withdrawal strategies on innate immunity markers and immunoglobulin (IgA, IgG, and IgM) levels in heat-stressed broiler chickens. The immunoglobulin levels exhibited significant differences among treatments. Compared with the control group, the IgA showed the highest level for the FWD-8 (1.59-fold), followed by FWD-12 (1.56-fold) and FWD-24 (1.39-fold). Moreover, the IgG level was significantly 1.8 to 2.0-fold higher for the FWD treatment groups. Meanwhile, the IgM level was significantly higher in FWD-24 and FWD-8 by 1.9 and 1.8-fold, respectively. On the other hand, serum pro-inflammatory cytokines, IL-1β and IFN-γ, significantly increased in the FWD groups compared with the control group by 8–9% and 49–61%, respectively. Furthermore, two distinguished innate immunity factors were significantly increased with FWD. Lysozyme and complement C3 protein showed a 26 to 27% and an 11 to 15% increase, respectively, in response to different FWD regimes compared to the control group. The results illustrate the significant positive impact of FWD on broilers’ immune and inflammation responses, with substantial progressive impacts on innate and humoral immunity in chronic heat-stressed broiler chickens.

## 4. Discussion

There is general agreement on the deleterious impact of heat stress on production performance parameters [3,13,18,43,44]. The average minimum and maximum recorded brooding temperature in the present study were 30 and 35 °C, respectively, which is considered the critical temperature zone (26–35 °C) for growing broilers [44,45]. Moreover, the current calculated THI values averaged from 29 to 33.8, which was classified as severe to very severe heat stress [28]. The premier factor for impaired performance of chickens is the drop in feed intake observed under heat stress. Other factors that can be attributed to performance deterioration under thermal challenge are impaired digestibility with the occurrence of physiological and metabolic changes that negatively influence feed efficiency [3,17]. Brugaletta et al. [46] stated that heat stress exposure stimulates tissue catabolism and subsequently weight loss in chickens. Mohamed et al. [47] introduced feed restrictions for three hours a day as an effective nutritional tool to improve production performance of broilers raised in hot climates. The present study also indicated significant improvement in heat-stressed broiler production performance parameters (i.e., BWG, FI, and FCR) with early feed withdrawal compared with the ad libitum feed group. Thus, early feed withdrawal seems to improve feed intake and re-establish the physiological homeostasis of birds, resulting in improving BWG, FI, and FCR.

Birds’ serum metabolites level can significantly be altered under heat stress and can be used as a physiological marker. Lu et al. [48] indicated a wide array of serum metabolites alterations in heat-stressed broilers. They reported a significant increase in serum urea, uric acid, and cholesterol in heat-stressed broilers. Lu, He, Ma, Zhang, Li, Jiang, Zhou, and Gao [48] linked the increase in serum urea and uric acid with protein degradation and muscle atrophy under heat stress. Heat stress was also reported to induce hypercholesterolemia in broiler chickens [49]. Meanwhile, a reduction in serum total cholesterol was achieved with feed restriction for three hours a day in heat-stressed broilers [47]. Moreover, the observed higher blood hemoglobin concentration in the FWD groups can be an additional physiological indicator of the achieved improvement in body condition and general physiological fitness of heat-stressed broilers [50]. Our results showed significantly higher serum Hb, TP, and globulin levels, with lower serum total cholesterol and uric acid for the FWD groups, which physiologically reflects the onset of metabolic adaptation to heat stress.

Another major piece of evidence that FWD induced physiological adaption was the significant reduction in leptin and the increase in thyroid hormone circulation levels, observed in the FWD groups. Leptin is an energy balance regulator hormone that plays a key role in the regulation of nutrient intake, and its high level suppresses feed intake in chickens [51,52]. Food deprivation or restriction was reported to induce a reduction in leptin circulation, causing a short-term reduction in energy expenditure and an increase in food intake [53]. Moreover, birds adapt to heat stress by lowering blood thyroid hormone levels, leading metabolic hormones to reduce metabolic heat production, which consequently reduces chickens’ performance [3]. Kpomasse et al. [54] stated that heat stress exposure induces a reduction in thyroid hormone levels in broilers. Beckford et al. [55] also reported a significant reduction in plasma T_3_ levels in heat-exposed broilers, with a substantial alteration in the hypothalamus–pituitary–thyroid axis. They concluded that chronic heat stress exposure induces a prolonged decrease in T_3_ circulation that contributes to the reduction in broilers’ growth rate. The reduction in the T_3_ circulation level under heat stress was reported to be associated with impaired production performance (i.e., final BW, BWG, FI, and FCR) [56]. A reduction in production performance and serum T_4_ was reported in broilers exposed to constant heat stress [57]. Based on the current observed reduction in leptin with the increase in the T_3_ and T_4_ hormone levels, it can be suggested that early feed withdrawal induced metabolic modulation, which improved production performance in heat-stressed broilers. Hence, the observed reduction in leptin circulation and the increase in T_3_ and T_4_ in the FWD groups can be the leading factor for the observed shift increase in feed intake that is directly reflected in a higher BWG and better feed efficiency.

An additional physiological marker demonstrating the onset of broiler thermotolerance adaptation induced by early feed withdrawal was the increasing level of HSP70 observed with the FWD treatments. HSP70 is one of HSPs families responsible for protecting various cellular processes and ensuring cells’ survival during stress [58]. Researchers demonstrated that HSP70 gene expression was associated with increasing the thermotolerance ability of native chicken breeds [59,60]. Furthermore, early feed restriction was reported to increase HSP70 levels in heat-stressed broilers [61]. Early feed restriction induced thermotolerance in broiler chickens, with an associated improvement in HSP70 response [18,58]. Goel, Ncho, Gupta, and Choi [8] stated that after early thermal manipulation, the up-regulation in HSP gene expression in chicks exposed to heat stress reflects the acquisition of thermotolerance. On the other hand, the negative effects of heat stress exposure on redox balance are well documented [62,63]. Heat stress was reported to induce oxidative stress that subsequently increases reactive oxygen species (ROS) formation and induces redox imbalance and immunosuppression [64]. In the present study, the control group exhibited lower antioxidant marker activity and higher lipid peroxidation levels. Al-Otaibi, Abdellatif, Al-Huwail, Abbas, Mehaisen, and Moustafa [49] reported a substantial reduction in total antioxidant capacity with an elevation in plasma MDA levels in laying hens subjected to heat stress. Alaqil, Abd El-Atty, and Abbas [56] also reported a significant 4.6-fold increase in MDA levels in broilers exposed to chronic heat stress compared with those reared under thermoneutral conditions. However, FWD improved the redox status, with a significant reduction in MDA levels and elevation in TAC and antioxidant enzyme activity. The favorable mitigation effect of FWD observed in our investigation can be justified by the improvement in antioxidant activity with the reduction in lipid peroxidation, which is reported to be directly related to broilers’ muscle quality and general growth [65,66].

Heat stress exposure is reported to induce immunosuppression in poultry [67,68]. Furthermore, immunosuppression with a significant reduction in immune cytokine levels, such as IL-1β, was reported in heat-stressed broilers [69]. Moreover, a significant drop in serum IgG and IgM concentrations was reported in two commercial broiler strains subjected to heat stress [7]. Korkmaz et al. [70] also reported a general suppression in immune functions with a reduction in IgA, IgG, and IgM in broilers subjected to heat stress. Currently, low levels of immune modulator cytokines, IgA, IgG, and IgM, were observed in the control group. Interestingly, a significant increase in pro-inflammatory cytokine secretion levels (i.e., IL-1β and IFN-γ) as well as an increase in serum globulin concentration and immunoglobulin levels was observed in response to FWD regimes. Interferon-gamma (IFN-γ) is a cytokine that plays a fundamental role in the regulation, maturation, and differentiation of cell-mediated immune responses in birds [71]. Saleh and Al-Zghoul [72] reported an increase in plasma and splenic gene expiration of IFN-γ in broilers subjected to acute heat stress and embryonic thermal adaptation, indicating the onset of heat tolerance acquisition. Thus, it can be inferred that the observed increase in the pro-inflammatory cytokines level illustrates thermotolerance adaptation in the FWD groups.

In addition to immune cells, the innate immune system uses a diverse range of soluble proteins to directly combat infections, identify pathogens, and trigger further immune responses [73,74]. The complement system is an important component of innate immunity, which fights infection by tagging pathogens for destruction, promoting inflammation, and directly killing cells. A key element in the avian innate immunity complement system is the complement component C3 protein that is up-regulated upon pro-inflammatory cytokine stimulation [74,75]. Lysozyme is an enzyme with antibacterial properties that is part of the innate immunity of animals [76]. Recently, exogenous lysozyme supplementation into broilers’ diets was reported as a promising antibiotic alternative growth promoter replacer as well as an immunomodulation agent [76,77,78]. Abdel-Latif, El-Hamid, Emam, Noreldin, Helmy, El-Far, and Elbestawy [77] reported a significant up-regulation of IFN-γ mRNA expression in lysozyme-supplemented broilers. This finding is consistent with our observation of increased lysozyme and IFN-γ levels in the FWD groups compared to the control. These results suggest a positive influence of FWD on key physiological processes related to both innate and humoral immune responses.

The current investigated FWD regimes seem to mitigate the undesirable impacts of heat stress on redox status and exhibit an immunomodulation effect. These effects can be justified by the onset of physiological adaptation to heat stress and induction of heat tolerance that restore the redox balance and subsequently immune activation [68]. Furthermore, the increased level of HSP70 with FWD beneficiary enhanced the immune responses as it was reported to boost infectious bursal disease (IBD) resistance in heat-stressed broilers [61]. Finally, early feed withdrawal proved to reverse the homeostatic and metabolic distresses induced by heat stress and appeared to be a promising approach to overcome this growing threat to the broiler industry’s sustainability.

## 5. Conclusions

It can be concluded from the present study that early feed withdrawal for 24 h, either continuously for one day or equally distributed over two or three days, can be applied as a non-invasive nutritional strategy to protect broiler chickens from the deleterious impacts of heat stress. Early feed withdrawal promotes thermotolerance development in broilers by inducing physiological changes that enhance performance under heat stress. Thus, aligned with the global rise in environmental temperatures, the currently proposed nutritional strategies holds promise for enhancing broilers’ production performance, strengthening their immune response, and maintaining redox balance when confronting challenging heat stress conditions.

## Figures and Tables

**Table 1:** Basal diet ingredients and chemical composition.

Ingredients (%)	Starter (0–21 Days)	Grower–Finisher (22–35 Days)
Yellow corn	54.00	58.93
Soybean meal, 44%	34.12	30.25
Corn gluten, 60%	6.10	4.9
Soy oil	1.00	1.18
Limestone	1.65	1.6
Monocalcium phosphate	1.65	1.65
Salt	0.45	0.45
Premix ^1^	0.30	0.30
DL-methionine, 98%	0.15	0.16
Lysine, HCl, 78%	0.30	0.30
NaCO_3_	0.28	0.28
**Chemical composition**		
Metabolizable energy, kcal/kg	2900	2951
Crude protein %	23.02	21.00
Ether extract %	3.48	3.78
Crude fiber %	3.64	3.47
Calcium %	0.99	0.96
Available phosphorus%	0.45	0.45
Lysine %	1.34	1.24
Methionine %	0.52	0.50
Threonine %	0.86	0.78

^1^ The premix provides the following vitamins (vit) and minerals per each kg of diet: vit A, 4550 IU; vit E, 7.5 IU; vit D3, 450 IU; vit K, 0.752 mg; vit B2, 3.75 mg; pantothenic acid, 3 mg; vit B3, 15.2 mg; vit B12, 0.006 mg; vit B7, 0.152 mg; folic acid, 0.376 mg; vit B1, 1.07 mg; pyridoxine, 3.78 mg; choline,157.5 g; Cu, 12 mg; I, 53 µg; Mn, 30.2 mg; Se, 90 µg; Zn, 53.0 mg; Fe, 67.8 mg.

**Table 2:** Weekly changes in ambient temperature, relative humidity (RH), and temperature–humidity index (THI).

Week of Age	Min Temp. (°C)	Max Temp. (°C)	RH (%)	Min THI ^1^	Max THI
1st	29.8	33.6	70.0	28.3	32.5
2nd	30.2	35.1	69.0	28.7	33.9
3rd	30.3	34.8	75.2	29.1	33.6
4th	30.4	35.4	75.6	29.2	34.3
5th	30.7	35.7	76.5	29.5	34.5
Mean	30.3	34.9	73.3	29.0	33.8

^1^ THI was calculated following the formula THI = Tdb − ((0.31–0.31 RH) (Tdb − 14.4)), where Tdb presents the dry bulb temperature in Celsius, and RH presents the relative humidity (%). The corresponding stress levels for the following THI values < 27.8, 27.8 to <28.9, 28.9 to <30.0, and ≥30 were identified as no stress, moderate, severe, and very severe heat stress, respectively [28]. °C: degree Celsius, min: minimum, max: maximum, Temp.: temperature.

**Table 3:** Production performance parameters of broiler chickens exposed to different early feed withdrawal strategies.

Items	Period	Treatments *				SEM	*p*-Value
		Control	FWD-24	FWD-12	FWD-8		
Body weight, g	Day 1	43.2	43.0	43.0	43.8	0.36	0.823
	Day 21	532 ^c^	566 ^a^	549 ^b^	551 ^b^	2.75	<0.0001
	Day 35	1841 ^d^	2201 ^a^	2011 ^c^	2085 ^b^	28.8	<0.0001
Body weight gain, g	Day 1 to 21	490 ^c^	524 ^a^	507 ^b^	508 ^b^	2.71	<0.0001
	Day 1 to 35	1799 ^d^	2158 ^a^	1969 ^c^	2042 ^b^	28.7	<0.0001
Feed intake, g	Day 1 to 21	699	684	688	681	3.33	0.230
	Day 22 to 35	2500 ^b^	2762 ^a^	2687 ^a^	2720 ^a^	124	<0.0001
	Day 1 to 35	3199 ^b^	3446 ^a^	3375 ^a^	3402 ^a^	23.9	<0.0001
FCR	Day 1 to 21	1.43 ^a^	1.30 ^c^	1.36 ^b^	1.34 ^bc^	0.11	<0.0001
	Day 1 to 35	1.78 ^a^	1.60 ^c^	1.72 ^b^	1.67 ^b^	0.02	<0.0001

Different superscript letters within a row denote significant differences (*p* ≤ 0.05). * Control: group with no feed withdrawal; FWD-24: feed withdrawal for 24 h on the 5th day of age; FWD-12: feed withdrawal for 12 h on the 3rd and the 5th day of age; FWD-8: feed withdrawal for 8 h on the 3rd, 4th, and 5th day of age. FCR: feed conversion ratio.

**Table 4:** Serum metabolic hormones and metabolites concentration of broilers affected by different early feed withdrawal strategies.

Items	Treatments ^1^				SEM	*p*-Value
	Control	FWD-24	FWD-12	FWD-8		
T_3_, ng/dL	3.92 ^c^	4.39 ^a^	4.13 ^b^	4.29 ^a^	0.04	<0.0001
T_4_, ng/dL	21.95 ^c^	24.73 ^a^	23.63 ^b^	23.87 ^b^	0.22	<0.0001
Leptin, ng/mL	2.18 ^a^	1.49 ^b^	1.59 ^b^	1.65 ^b^	0.08	0.003
Total protein, g/dL	5.05 ^b^	6.25 ^a^	5.95 ^a^	6.13 ^a^	0.11	<0.0001
Albumin, g/dL	2.58	3.02	2.82	2.98	0.08	0.244
Globulin, g/dL	2.47 ^b^	3.23 ^a^	3.13 ^a^	3.15 ^a^	0.08	<0.0001
T-chol, mg/dL	212.3 ^a^	191.7 ^b^	196.8 ^b^	194.8 ^b^	2.00	<0.0001
Uric acid, mg/dL	4.52 ^a^	4.02 ^b^	4.08 ^b^	4.05 ^b^	0.07	0.014
Hb, g/dL	10.01 ^b^	11.19 ^a^	10.99 ^a^	11.11 ^a^	0.12	<0.0001

Different superscript letters within a row denote significant differences (*p* ≤ 0.05). ^1^ Control: group with no feed withdrawal; FWD-24: feed withdrawal for 24 h on the 5th day of age; FWD-12: feed withdrawal for 12 h on the 3rd and the 5th day of age; FWD-8: feed withdrawal for 8 h on the 3rd, 4th, and 5th day of age. T-chol: total cholesterol, Hb: hemoglobin.

**Table 5:** Liver HSP70 and serum oxidation markers of broilers affected by different early feed withdrawal strategies.

Parameters	Treatments *				SEM	*p*-Value
	Control	FWD-24	FWD-12	FWD-8		
HSP70, ng/mg	2.89 ^b^	5.13 ^a^	5.03 ^a^	5.07 ^a^	0.21	<0.0001
TAC, U/mL	10.52 ^b^	13.32 ^a^	12.97 ^a^	13.18 ^a^	0.28	<0.0001
CAT, U/mL	2.90 ^b^	5.58 ^a^	5.46 ^a^	5.23 ^a^	0.26	<0.0001
SOD, U/mL	134.9 ^b^	151.2 ^a^	149.6 ^a^	148.3 ^a^	1.77	<0.0001
MDA, nmol/mL	5.28 ^a^	2.63 ^b^	2.98 ^b^	2.93 ^b^	0.25	<0.0001

Different superscript letters within a row denote significant differences (*p* ≤ 0.05). TAC: total antioxidant capacity; CAT: catalase; SOD: superoxide dismutase; MDA: malondialdehyde; SEM: standard error of mean. * Control: group with no feed withdrawal; FWD-24: feed withdrawal for 24 h on the 5th day of age; FWD-12: feed withdrawal for 12 h on the 3rd and the 5th day of age; FWD-8: feed withdrawal for 8 h on the 3rd, 4th, and 5th day of age.

**Table 6:** Serum levels of immunoglobulin, pro-inflammatory cytokines, and innate immunity factors of broilers affected by different early feed withdrawal strategies.

Parameters	Treatments *				SEM	*p*-Value
	Control	FWD-24	FWD-12	FWD-8		
IgA, mg/100 mL	3.33 ^c^	4.64 ^b^	5.21 ^ab^	5.29 ^a^	0.19	<0.0001
IgG, mg/100 mL	1.96 ^b^	3.95 ^a^	3.47 ^a^	3.60 ^a^	0.19	<0.0001
IgM, mg/100 mL	0.96 ^b^	1.79 ^a^	1.31 ^ab^	1.69 ^a^	0.10	0.010
IL-1β, µg/mL	149.50 ^b^	162.33 ^a^	163.67 ^a^	163.33 ^a^	2.02	0.023
IFN-γ, pg/mL	7.28 ^c^	10.87 ^b^	11.32 ^ab^	11.77 ^a^	0.39	<0.001
Lysozyme, µg/mL	134.83 ^b^	169.67 ^a^	170.17 ^a^	171.33 ^a^	3.50	<0.001
Complement C3, g/L	1.10 ^b^	1.23 ^a^	1.22 ^a^	1.26 ^a^	0.01	<0.001

Different superscript letters within a row denote significant differences (*p* ≤ 0.05). * Control: group with no feed withdrawal; FWD-24: feed withdrawal for 24 h on the 5th day of age; FWD-12: feed withdrawal for 12 h on the 3rd and the 5th day of age; FWD-8: feed withdrawal for 8 h on the 3rd, 4th, and 5th day of age. IL-1β: interleukin 1 beta; INF-γ: interferon gamma; Ig: immunoglobulin.

## Data Availability

The datasets analyzed in the current study are available from the corresponding author on reasonable request.

## References

[1] Al-Khalaifah H., Al-Nasser A. Using native plants in poultry feed: Food security and sustainability approach. Proceedings of the 23rd International Multidisciplinary Scientific GeoConference SGEM 2023.

[2] Kamel N.N., Ahmed A.M.H., Mehaisen G.M.K., Mashaly M.M., Abass A.O. (2017). Depression of leukocyte protein synthesis, immune function and growth performance induced by high environmental temperature in broiler chickens. Int. J. Biometeorol..

[3] Onagbesan O.M., Uyanga V.A., Oso O., Tona K., Oke O.E. (2023). Alleviating heat stress effects in poultry: Updates on methods and mechanisms of actions. Front. Vet. Sci..

[4] Rostagno M.H. (2020). Effects of heat stress on the gut health of poultry. J. Anim. Sci..

[5] Yin C., Tang S.L., Liu L., Cao A.Z., Xie J.J., Zhang H.F. (2021). Effects of bile acids on growth performance and lipid metabolism during chronic heat stress in broiler chickens. Animals.

[6] Alzarah M.I., Althobiati F., Abbas A.O., Mehaisen G.M.K., Kamel N.N. (2021). Citrullus colocynthis seeds: A potential natural immune modulator source for broiler reared under chronic heat stress. Animals.

[7] Awad E.A., Najaa M., Zulaikha Z.A., Zulkifli I., Soleimani A.F. (2020). Effects of heat stress on growth performance, selected physiological and immunological parameters, caecal microflora, and meat quality in two broiler strains. Asian-Australas. J. Anim. Sci..

[8] Goel A., Ncho C.M., Gupta V., Choi Y.H. (2023). Embryonic modulation through thermal manipulation and in ovo feeding to develop heat tolerance in chickens. Anim. Nutr..

[9] Oke O.E., Alo E.T., Oke F.O., Oyebamiji Y.A., Ijaiya M.A., Odefemi M.A., Kazeem R.Y., Soyode A.A., Aruwajoye O.M., Ojo R.T. (2020). Early age thermal manipulation on the performance and physiological response of broiler chickens under hot humid tropical climate. J. Therm. Biol..

[10] Zineb B., Said D., Djilali B. (2021). Impact of both early-age acclimation and linseed dietary inclusion on fat deposition and fatty acids’ meat traits in heat-stressed broiler chickens. J. Adv. Vet. Anim. Res..

[11] Ahmad R., Yu Y.H., Hsiao F.S., Su C.H., Liu H.C., Tobin I., Zhang G., Cheng Y.H. (2022). Influence of heat stress on poultry growth performance, intestinal inflammation, and immune function and potential mitigation by probiotics. Animals.

[12] Ogbuagu N.E., Ayo J.O., Aluwong T., Akor-Dewu M.B. (2023). Effect of L-serine on circadian variation of cloacal and body surface temperatures in broiler chickens subjected to feed restriction during the hot-dry season. J. Therm. Biol..

[13] Bilal R.M., Hassan F.U., Farag M.R., Nasir T.A., Ragni M., Mahgoub H.A.M., Alagawany M. (2021). Thermal stress and high stocking densities in poultry farms: Potential effects and mitigation strategies. J. Therm. Biol..

[14] Choi J., Kong B.Y.W., Bowker B.C., Zhuang H., Kim W.K. (2023). Nutritional strategies to improve meat quality and composition in the challenging conditions of broiler production: A review. Animals.

[15] Rahman, Hidayat C. (2020). Reducing negative effect of heat stress in broiler through nutritional and feeding strategy. IOP Conf. Ser. Earth Environ. Sci..

[16] Saracila M., Panaite T.D., Papuc C.P., Criste R.D. (2021). Heat stress in broiler chickens and the effect of dietary polyphenols, with special reference to Willow (*Salix* spp.) bark supplements—A review. Antioxidants.

[17] Teyssier J.R., Brugaletta G., Sirri F., Dridi S., Rochell S.J. (2022). A review of heat stress in chickens. Part II: Insights into protein and energy utilization and feeding. Front. Physiol..

[18] Wasti S., Sah N., Mishra B. (2020). Impact of heat stress on poultry health and performances, and potential mitigation strategies. Animals.

[19] Al-Surrayai T., Al-Khalaifah H. (2022). Dietary supplementation of fructooligosaccharides enhanced antioxidant activity and cellular immune response in broiler chickens. Front. Vet. Sci..

[20] Attia Y.A., Al-Khalaifah H., Abd El-Hamid H.S., Al-Harthi M.A., Alyileili S.R., El-Shafey A.A. (2022). Antioxidant status, blood constituents and immune response of broiler chickens fed two types of diets with or without different concentrations of active yeast. Animals.

[21] El-Moniary M.M., Hemid A.A., El-Wardany I., Gehad A., Gouda A. (2010). The effect of early age heat conditioning and some feeding programs for heat-stressed broiler chicks on: 1—Productive performance. World J. Agric. Sci..

[22] Farghly M.F.A., Mahrose K.M., Galal A.E., Ali R.M., Ahmad E.A.M., Rehman Z.U., Ullah Z., Ding C. (2018). Implementation of different feed withdrawal times and water temperatures in managing turkeys during heat stress. Poult. Sci..

[23] Nawaz A.H., Amoah K., Leng Q.Y., Zheng J.H., Zhang W.L., Zhang L. (2021). Poultry response to heat stress: Its physiological, metabolic, and genetic implications on meat production and quality including strategies to improve broiler production in a warming world. Front. Vet. Sci..

[24] Lin H., Jiao H., Buyse J., Decuypere E. (2006). Strategies for preventing heat stress in poultry. Worlds Poult. Sci. J..

[25] Zhan X.A., Wang M., Ren H., Zhao R.Q., Li J.X., Tan Z.L. (2007). Effect of early feed restriction on metabolic programming and compensatory growth in broiler chickens. Poult. Sci..

[26] National Research Council (1994). Nutrient Requirements of Poultry: 9th Revised Edition.

[27] Marai I.F.M., Ayyat M.S., Abd El-Monem U.M. (2001). Growth performance and reproductive traits at first parity of New Zealand White female rabbits as affected by heat stress and its alleviation under Egyptian conditions. Trop. Anim. Health Prod..

[28] Dedousi A., Kritsa M.Z., Sossidou E.N. (2023). Thermal comfort, growth performance and welfare of olive pulp fed broilers during hot season. Sustainability.

[29] Jain N.C. (1986). Schalm’s Veterinary Hematology.

[30] Sánchez-Carbayo M., Mauri M., Alfayate R., Miralles C., Soria F. (1999). Analytical and clinical evaluation of TSH and thyroid hormones by electrochemiluminescent immunoassays. Clin. Biochem..

[31] Weichselbaum T.E. (1946). An accurate and rapid method for the determination of proteins in small amounts of blood serum and plasma. Am. J. Clin. Pathol..

[32] Doumas B.T., Biggs H.G., Arends R.L., Pinto P.V.C., Cooper G.R. (1972). Determination of serum albumin. Standard Methods of Clinical Chemistry.

[33] Allain C.C., Poon L.S., Chan C.S., Richmond W., Fu P.C. (1974). Enzymatic determination of total serum cholesterol. Clin. Chem..

[34] Sanders G.T., Pasman A.J., Hoek F.J. (1980). Determination of uric acid with uricase and peroxidase. Clin. Chim. Acta.

[35] Janaszewska A., Bartosz G. (2002). Assay of total antioxidant capacity: Comparison of four methods as applied to human blood plasma. Scand. J. Clin. Lab. Investig..

[36] Aebi H. (1984). Catalase in vitro. Methods Enzymol..

[37] Sun Y., Oberley L.W., Li Y. (1988). A simple method for clinical assay of superoxide dismutase. Clin. Chem..

[38] Placer Z.A., Cushman L.L., Johnson B.C. (1966). Estimation of product of lipid peroxidation (malonyl dialdehyde) in biochemical systems. Anal. Biochem..

[39] Tiemann U., Brüssow K.P., Jonas L., Pöhland R., Schneider F., Dänicke S. (2006). Effects of diets with cereal grains contaminated by graded levels of two Fusarium toxins on selected immunological and histological measurements in the spleen of gilts. J. Anim. Sci..

[40] Lie Ø., Syed M., Solbu H. (1986). Improved agar plate assays of bovine lysozyme and haemolytic complement activity. Acta Vet. Scand..

[41] Gouda A., Tolba S., Mahrose K., Felemban S.G., Khafaga A.F., Khalifa N.E., Jaremko M., Moustafa M., Alshaharni M.O., Algopish U. (2024). Heat shock proteins as a key defense mechanism in poultry production under heat stress conditions. Poult. Sci..

[42] Anderson R.L., Wang C.Y., van Kersen I., Lee K.J., Welch W.J., Lavagnini P., Hahn G.M. (1993). An immunoassay for heat shock protein 73/72: Use of the assay to correlate HSW3/72 levels in mammalian cells with heat response. Int. J. Hyperth..

[43] Biswal J., Vijayalakshmy K., Bhattacharya T.K., Rahman H. (2022). Impact of heat stress on poultry production. Worlds Poult. Sci. J..

[44] Shakeri M., Oskoueian E., Le H.H., Shakeri M. (2020). Strategies to combat heat stress in broiler chickens: Unveiling the roles of selenium, vitamin E and vitamin C. Vet. Sci..

[45] Fathi M.M., Galal A., Radwan L.M., Abou-Emera O.K., Al-Homidan I.H. (2022). Using major genes to mitigate the deleterious effects of heat stress in poultry: An updated review. Poult. Sci..

[46] Brugaletta G., Teyssier J.R., Rochell S.J., Dridi S., Sirri F. (2022). A review of heat stress in chickens. Part I: Insights into physiology and gut health. Front. Physiol..

[47] Mohamed A.S.A., Lozovskiy A.R., Ali A.M.A. (2019). Strategies to combat the deleterious impacts of heat stress through feed restrictions and dietary supplementation (vitamins, minerals) in broilers. J. Indones. Trop. Anim. Agric..

[48] Lu Z., He X., Ma B., Zhang L., Li J., Jiang Y., Zhou G., Gao F. (2018). Serum metabolomics study of nutrient metabolic variations in chronic heat-stressed broilers. Br. J. Nutr..

[49] Al-Otaibi M.I.M., Abdellatif H.A.E., Al-Huwail A.K.A., Abbas A.O., Mehaisen G.M.K., Moustafa E.S. (2022). Hypocholesterolemic, antioxidative, and anti-Inflammatory effects of dietary Spirulina platensisis supplementation on laying hens exposed to cyclic heat stress. Animals.

[50] Minias P. (2015). The use of haemoglobin concentrations to assess physiological condition in birds: A review. Conserv. Physiol..

[51] Taouis M., Dridi S., Cassy S., Benomar Y., Raver N., Rideau N., Picard M., Williams J., Gertler A. (2001). Chicken leptin: Properties and actions. Domest. Anim. Endocrinol..

[52] Murugesan S., Nidamanuri A.L. (2022). Role of leptin and ghrelin in regulation of physiological functions of chicken. World’s Poult. Sci. J..

[53] Boswell T. (2005). Regulation of energy balance in birds by the neuroendocrine hypothalamus. J. Poult. Sci..

[54] Kpomasse C.C., Oke O.E., Houndonougbo F.M., Tona K. (2021). Broilers production challenges in the tropics: A review. Vet. Med. Sci..

[55] Beckford R.C., Ellestad L.E., Proszkowiec-Weglarz M., Farley L., Brady K., Angel R., Liu H.C., Porter T.E. (2020). Effects of heat stress on performance, blood chemistry, and hypothalamic and pituitary mRNA expression in broiler chickens. Poult. Sci..

[56] Alaqil A.A., Abd El-Atty H.K., Abbas A.O. (2022). Intermittent lighting program relieves the deleterious effect of heat stress on growth, stress biomarkers, physiological status, and immune response of broiler chickens. Animals.

[57] Li X.M., Zhang M.H., Feng J.H., Zhou Y. (2021). Myostatin and related factors are involved in skeletal muscle protein breakdown in growing broilers exposed to constant heat stress. Animals.

[58] Balakrishnan K.N., Ramiah S.K., Zulkifli I. (2023). Heat shock protein response to stress in poultry: A review. Animals.

[59] Asadollahpour Nanaei H., Kharrati-Koopaee H., Esmailizadeh A. (2022). Genetic diversity and signatures of selection for heat tolerance and immune response in Iranian native chickens. BMC Genom..

[60] Radwan L.M. (2020). Genetic improvement of egg laying traits in Fayoumi chickens bred under conditions of heat stress through selection and gene expression studies. J. Therm. Biol..

[61] Liew P.K., Zulkifli I., Hair-Bejo M., Omar A.R., Israf D.A. (2003). Effects of early age feed restriction and heat conditioning on heat shock protein 70 expression, resistance to infectious bursal disease, and growth in male broiler chickens subjected to heat stress. Poult. Sci..

[62] Khan R.U., Naz S., Ullah H., Ullah Q., Laudadio V., Qudratullah, Bozzo G., Tufarelli V. (2023). Physiological dynamics in broiler chickens under heat stress and possible mitigation strategies. Anim. Biotechnol..

[63] Oke O.E., Uyanga V.A., Iyasere O.S., Oke F.O., Majekodunmi B.C., Logunleko M.O., Abiona J.A., Nwosu E.U., Abioja M.O., Daramola J.O. (2021). Environmental stress and livestock productivity in hot-humid tropics: Alleviation and future perspectives. J. Therm. Biol..

[64] Lauridsen C. (2019). From oxidative stress to inflammation: Redox balance and immune system. Poult. Sci..

[65] Kuttappan V.A., Manangi M., Bekker M., Chen J.X., Vazquez-Anon M. (2021). Nutritional intervention strategies using dietary antioxidants and organic trace minerals to reduce the incidence of wooden breast and other carcass quality defects in broiler birds. Front. Physiol..

[66] Zhao Y.Y., Li Z., Wang X.C., Zhao F., Wang C., Zhang Q.Y., Chen X.Y., Geng Z.Y., Zhang C. (2022). Resveratrol attenuates heat stress-induced impairment of meat quality in broilers by regulating the Nrf2 signaling pathway. Animals.

[67] Hirakawa R., Nurjanah S., Furukawa K., Murai A., Kikusato M., Nochi T., Toyomizu M. (2020). Heat stress causes immune abnormalities via massive damage to effect proliferation and differentiation of lymphocytes in broiler chickens. Front. Vet. Sci..

[68] Zmrhal V., Svoradova A., Venusova E., Slama P. (2023). The influence of heat stress on chicken immune system and mitigation of negative impacts by baicalin and baicalein. Animals.

[69] Tang L.P., Li W.H., Liu Y.L., Lun J.C., He Y.M. (2021). Heat stress inhibits expression of the cytokines, and NF-κB-NLRP3 signaling pathway in broiler chickens infected with salmonella typhimurium. J. Therm. Biol..

[70] Korkmaz D., Kum S., Eren U. (2023). Effects of vitamin E on T cell subsets and immunoglobulin-containing plasma cells in the spleen of heat-stressed broiler chickens. Med. Weter.-Vet. Med.-Sci. Pract..

[71] Wigley P., Kaiser P. (2003). Avian cytokines in health and disease. Braz. J. Poult. Sci..

[72] Saleh K.M.M., Al-Zghoul M.B. (2019). Effect of acute heat stress on the mRNA levels of cytokines in broiler chickens subjected to embryonic thermal manipulation. Animals.

[73] Veldhuizen E.J.A., Dalgaard T.S., Kaspers B., Schat K.A., Göbel T.W., Vervelde L. (2022). Chapter 8.5—Soluble components and acute-phase proteins. Avian Immunology.

[74] Juul-Madsen H.R., Viertlböeck B., Härtle S., Smith A.L., Göbel T.W., Schat K.A., Kaspers B., Kaiser P. (2014). Chapter 7—Innate Immune Responses. Avian Immunology.

[75] Juul-Madsen H.R., Viertlboeck B., Smith A.L., Göbel T.W.F., Davison F., Kaspers B., Schat K.A. (2008). 7-Avian innate immune responses. Avian Immunology.

[76] Ferraboschi P., Ciceri S., Grisenti P. (2021). Applications of lysozyme, an innate immune defense factor, as an alternative antibiotic. Antibiotics.

[77] Abdel-Latif M.A., El-Hamid H.S.A., Emam M., Noreldin A.E., Helmy Y.A., El-Far A.H., Elbestawy A.R. (2024). Dietary lysozyme and avilamycin modulate gut health, immunity, and growth rate in broilers. BMC Vet. Res..

[78] Bastamy M., Raheel I., Elbestawy A., Diab M., Hammad E., Elebeedy L., El-Barbary A.M., Albadrani G.M., Abdel-Daim M.M., Abdel-Latif M.A. (2024). Postbiotic, anti-inflammatory, and immunomodulatory effects of aqueous microbial lysozyme in broiler chickens. Anim. Biotechnol..

